# Economic Burden of Neonatal Intensive Care Unit Admissions in Public Hospitals of Sidama Regional State, Ethiopia, 2024

**DOI:** 10.1155/ijpe/5831674

**Published:** 2026-11-15

**Authors:** Meskerem Jisso Ebido, Sisay Dejene, Yemisrach shiferaw, Yusuf Haji, Dubale Dulla, Achamyelesh Gebretsadik

**Affiliations:** ^1^ School of Public Health College of Medicine and Health Sciences Hawassa University Hawassa Sidama Regional State Ethiopia hu.edu.et; ^2^ Department of Midwifery College of Medicine and Health Sciences Hawassa University Hawassa Sidama Regional State Ethiopia hu.edu.et

**Keywords:** economic burden, neonatal intensive care unit, out-of-pocket expenses

## Abstract

**Background:**

Neonatal intensive care unit (NICU) admission imposes a significant economic burden on parents, particularly in low‐resource settings such as Ethiopia. Understanding the financial impact of NICU care is of paramount importance for improving healthcare delivery and reducing out‐of‐pocket expenses for parents. The aim of this study was to assess the economic burden of NICU admissions and associated factors in public hospitals in Sidama Regional State, Ethiopia.

**Methods:**

A total of 446 parents of neonate admitted to NICUs in six government hospitals in Sidama regional state. A cross‐sectional study design was executed. Structured questionnaire and medical record reviews were used to collect the data. Expenses of the parents were categorized in to direct cost like medicine and laboratory test and indirect costs like food, transportation, and others. Regression analysis was applied to identify factors associated with the overall economic burden.

**Results:**

Median total cost of NICU admission was 2007 ETB ($34.76), of this the indirect costs was account for 67.8%. The highest portion 81.8% of the indirect costs was constituted by food expense. Medicine cost accounted 54.4% of the direct costs which was the highest. More than half of the parents (51.3%) covered costs using their current income, while 24.7% resorted to borrowing. Duration of hospital stay (adjusted exp(*β*) = 1.02, 95% CI: 1.01–1.03) was significantly associated with higher economic burden, while admission to a general hospital, relative to a primary hospital, was significantly associated with lower economic burden (adjusted exp(*β*) = 0.21, 95% CI: 0.14–0.99).

**Conclusion:**

Economic burden of NICU admissions is substantial, with indirect costs, particularly food expenses, being the largest contributor. Prolonged hospital stays and admission to primary hospitals were associated with higher costs. Interventions to reduce out‐of‐pocket expenses, such as improving access to medical insurance and strengthening primary healthcare facilities, are recommended.

## 1. Introduction

Particularly in developing nations, the cost of neonatal intensive care unit (NICU) admission poses a serious challenge to families and healthcare systems alike. As result to the direct and indirect costs of NICU admission, having a baby at NICU is very costly for the parents. Lost income as a result of giving care duties, transportation, and food expenses is included as indirect costs, and cost of health care, including medicine, laboratory tests, and hospital stays, is categorized as direct cost [1, 2].

Parents in low‐resource settings, including Ethiopia, face greater financial strain from NICU admissions. Parents are extremely affected by out‐of‐pocket costs, and many are compelled to take out loans, liquidate assets, or cut back on household spending to cover essential care [3]. There is also considerable variation in the median treatment cost for a newborn admitted to a NICU across facilities and countries: For instance, a study in Nepal reported a mean cost of about $33.2 [4], whereas a Brazilian study reported costs as high as $2328 [5]. This variation likely reflects differences in healthcare financing structures, insurance coverage, and income levels across settings, underscoring the need for context‐specific solutions to address the financial burden faced by families.

As the result of the expense of NICU care, families usually face financial hardship, which can fuel stress and anxiety [6]. Financial hardships have been demonstrated to worsen parental dissatisfaction and mental health issues [7]. In addition, financial strains can make families to feel powerless which in turn make difficult to them to give the care they needs to their newborns [8].

Furthermore, the effect of financial stress extends more than the immediate expenses occurred during the medical treatment. In long‐term financial strains affects, the quality of life of the family′s and the long‐run access to health care services can result from accumulated debt or diminished saving [9]. As a result, developing strategies to improve health care delivery and family support requires an understanding of economic burden of NICU stays.

The true costs and household financial burden in Ethiopia, especially in the Sidama Regional State, are not well documented, despite the increased awareness of the financial difficulties associated with neonatal care. Policies that reduce financial barriers to care and enhance neonatal health outcomes require an understanding of the economic burden of NICU admissions.

The aim of this study is to estimate the cost of NICU admission in public Sidama Regional State hospitals, Ethiopia, and identify the factors associated with high cost.

## 2. Methods

### 2.1. Study Design and Setting

A cross‐sectional study was conducted in six public hospitals in Sidama Regional State, Ethiopia, from April 2023 to March 2024. The region has one referral hospital, three general hospitals, and 13 district hospitals. The study included parents of neonates admitted to the NICU for a minimum of 3 days. Ethiopia is classified as a low‐income country, with a per‐capita gross national income (GNI) of approximately $1020 in 2023, equivalent to roughly $85 per month [10].

### 2.2. Study Population and Sampling

The study population consisted of parents of neonates admitted to the NICU in selected hospitals. A multistage sampling technique was used to select hospitals and participants. Six hospitals (one referral, three general, and two district hospitals) were randomly selected, and the required minimum sample size was calculated using single population proportion formula assuming 50% of the burden proportion and 384 were the calculated sample size; however, given that this study was conducted as part of a larger parent research project, a larger sample of 446 parents was enrolled.

### 2.3. Data Collection

Data were collected using a structured interviewer‐administered questionnaire and medical record reviews. The questionnaire captured information on direct costs (e.g., medications, laboratory tests, and bed fees) and indirect costs (e.g., transportation, food, and lost productivity). Costs were recorded in Ethiopian Birr (ETB) and converted to USD using the exchange rate of 57.73 ETB/USD (as of June 26, 2024). The full questionnaire, including its sections on direct and indirect cost items, has been attached as Supporting Information to support replication. In this study, financial burden was operationalized as the combination of direct medical costs, indirect costs (e.g., transportation, food, and lost productivity), and the coping strategies (e.g., borrowing and asset sales) that parents used to meet these costs, consistent with the multicomponent conceptualization of financial burden described by Lambiase et al. [11].

### 2.4. Data Analysis

Data were entered into KoBo and analyzed using SPSS Version 27. Descriptive statistics were used to summarize costs, and linear regression analysis was employed to identify factors associated with the total economic burden. Costs were log‐transformed due to nonnormal distribution, and coefficients were exponentiated for interpretation. Bivariate regression was first used to assess the association of each candidate independent variable (neonate age, admission type, admission reason, type of hospital, presence of insurance, total hospital stay, residence location, gestational age, sex of neonate, and number of comorbidities at admission) with the total cost of treatment. All variables that were significantly associated with the outcome at the bivariate analysis stage (*p* < 0.05) were entered simultaneously into the multivariable regression model using the Enter method in SPSS. Four variables met this criterion and were retained for the multivariable model: reason for admission, type of hospital, hospital stay, and gestational age of neonate. In the multivariable analysis, type of hospital and hospital stay were found to significantly influence the overall economic cost at a *p* value of less than 0.05. During the collection time, financial hardship was taken when people pay their medical bills by borrowing, selling assets, contribution from friends or relatives, reducing basic household consumption, or combination of them.

## 3. Results

### 3.1. Sociodemographic Characteristics of Respondents

Four hundred forty‐six respondents were enrolled in this study with response rate of 100%.

The respondents mean age (±standard deviation [SD]) was 26.46 (±5.01) years. Nearly one‐fourth of respondents (24.7%) were found in the age group of greater or equal to 30 years. Three hundred seventeen (71.1%) of respondents were female by their gender. One hundred thirty‐six (30.5%), nearly half (48.0%), and 172 (38.6%) of respondents were held college and above level education, housewife, and rural dwellers, respectively. With respect to marital status, most (93.0%) of respondents were married (Table 1).

**Table 1:** Sociodemographic characteristics of respondents at public hospitals of Sidama region, Ethiopia, 2024.

Variables	Categories	Frequency	Percentage (%)
Parents age (in completed years)	≤ 24	145	32.5
	25–29	191	42.8
	≥ 30	110	24.7

Parent′s sex	Male	129	28.9
	Female	317	71.1

Educational status	No education	26	5.8
	Primary	100	22.4
	Secondary	184	41.3
	College and above	136	30.5

Marital status	Married	415	93.0
	Never married	10	2.2
	Divorced/separated/widowed	21	4.7

Occupational status	Housewife	214	48.0
	Government employee	40	9.0
	Private employee	145	32.5
	Others^a^	47	10.5

Place of residence	Rural	172	38.6
	Semiurban	126	28.3
	Urban	148	33.2

^a^Daily laborer, farmer, and student.

### 3.2. The Economic Burden of Neonate Admitted to NICU Ward

The median total cost of treatment for a neonate admitted to the NICU was 2007 ETB, with an interquartile range (IQR) of 1420–2737 ETB. Indirect costs accounted for the largest portion of the total cost at 67.8%, while direct costs made up the remaining 32.2% (Table 2). The direct costs included expenses for medicine, laboratory tests, bed usage, imaging, and incubator use. Among these, medicine (54.4%) and bed costs (30.3%) constituted the majority of the direct costs. Regarding indirect costs, food accounted for approximately 81.8%, followed by transportation (11.6%) and productivity loss/work time loss due to the hospital stay (6.6%) (Figure 1). More than half of the parents, 229 (51.3%), covered the total cost of their neonate′s admission to the NICU from their current income, and 241 (54%) used their savings. Additionally, 47 parents (10.5%) covered the treatment costs by selling assets, and 110 parents (24.7%) did so by borrowing.

**Table 2:** The median cost of treatment for neonate admitted in NICU ward.

Treatment cost for neonate admitted to NICU ward	Median (IQR)	
	ETB	USD^a^
Total cost	2007 (1420–2737)	34.76 (24.60–47.41)
Direct cost	471 (208–1050)	8.16 (3.60–18.19)
Indirect cost	1307 (820–1909)	22.64 (14.20–33.07)

^a^Used the exchange rate of 57.73 (based on the national bank exchange rate of June 26, 2024).

**Figure 1:**
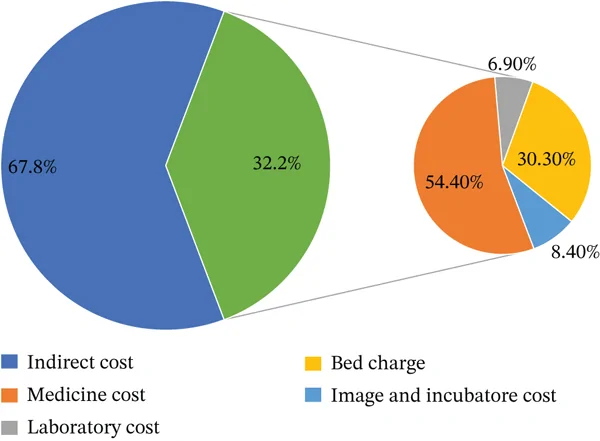
Distribution of direct and indirect cost.

Since the outcome variables (total cost) were nonnormal, we log‐transformed the data before we fitted the linear regression models. We calculated the coefficients of the dependent variable in a bid to interpret the results of the bivariable and multivariable regression models.

The economic cost for neonates admitted to general hospitals was 79% lower relative to primary hospitals (adjusted exp(*β*) = 0.21, 95% CI: 0.14–0.99). In addition, each additional day of hospital stay was associated with a 2% increase in the total cost of treatment (adjusted exp(*β*) = 1.02, 95% CI: 1.01–1.03) (Table 3).

**Table 3:** Multivariate regression analysis of factors associated with the total economic burden of neonate admitted to NICU ward.

Variables	Univariate analysis		Multivariate analysis	
	exp(*β*) (95% CI)^a^	*p* value	exp(*β*) (95% CI)^a^	*p* value
Age	1.00 (0.99–1.01)	0.582	0.99 (0.98–1.00)	0.079
Reason for admission				
Asphyxia	1.08 (0.96–1.21)	0.166	1.15 (0.98–1.33)	0.069
Jaundice	1.04 (0.92–1.18)	0.541	1.12 (0.96–1.32)	0.133
Sepsis	0.91 (0.81–1.01)	0.078	1.05 (0.90–1.22)	0.506
Prematurity	1.11 (0.98–1.26)	0.080	0.95 (0.70–1.28)	0.748
Respiratory distress	0.95 (0.84–1.08)	0.514	1.06 (0.91–1.25)	0.408
Congenital anomaly	Ref		Ref	
Hospital level	Ref		Ref	
Primary	0.80 (0.70–0.92)	0.002	**0.21 (0.14–0.99)**	**0.036**
General	1.23 (1.07–1.42)	0.002	1.14 (0.88–1.33)	0.553
Specialized				
Have medical insurance				
Yes	0.96 (0.87–1.06)		0.99 (0.89–1.09)	0.885
No	Ref		Ref	
Length of stay in the hospital	1.22 (1.11–1.35)	< 0.0001	**1.02 (1.01–1.03)**	**0.005**
Gestational age				
Term	Ref		Ref	
Preterm	1.12 (0.99–1.26)		1.16 (0.88–1.52)	0.283
Post term	0.93 (0.73–1.18)		0.95 (0.74–1.24)	0.747
Sex of neonate				
Male	1.04 (0.94–1.15)	0.415	1.06 (0.96–1.18)	0.218
Female	Ref		Ref	
Number of comorbidities during admission	Ref		Ref	
1	0.96 (0.85–1.08)	0.574	0.88 (0.74–1.04)	0.144
2	0.91 (0.74–1.10)	0.332	0.78 (0.58–1.05)	0.104
≥ 3				

*Note:* The bold values represent significant variables.

Abbreviation: ref = reference category.

^a^Values are exponentiated regression coefficients, exp(*β*) (i.e., the multiplicative change in total cost), with 95% CI, for both the univariate and multivariate models.

## 4. Discussion

The findings of this study reveal the substantial economic burden of NICU admissions on families in Sidama Regional State, Ethiopia. The total median expense of 2007 ETB ($34.76) is significant, particularly relative to the low national average monthly income reported in the Methods section [10]. Indirect expenses, which were largely driven by transport and food, comprised the highest percentage of expenses, indicating the hidden financial costs incurred by families in seeking neonatal care. This agrees with studies in Nepal and Nigeria, where indirect costs were also the main component of the economic burden [2]. Hospital stay was one of the strongest predictors of total economic cost, with each additional day increasing the cost by 2%. This concurs with studies in the United States and Nepal, where hospital stays of longer duration were also significantly associated with increased cost [4, 5, 9]. Hospitalization for extended periods not only increases direct medical expenses but also compounds indirect expenses, such as loss of productivity and other caregiving needs.

The hospital level also significantly influenced costs, with lower costs being incurred in general hospitals compared to primary hospitals [12, 13]. This may be due to higher availability of resources and more efficient provision of care in general hospitals that reduces the need for external referrals or additional treatments that are costly [12, 14, 15]. The finding also indicates inequity in the distribution of resources and capacity across levels of healthcare facilities that must be addressed in ensuring equitable access to affordable neonatal care.

The coping strategies used by families, such as sale of assets and borrowing, point to the severe financial burden exacted by NICU stays. These actions can have long‐term consequences, including debt and reduced capacity of households to absorb future economic shocks [3]. The high reliance on out‐of‐pocket expenditures underscores the need for policies that protect families from catastrophic health expenditures, such as the extension of health insurance coverage and the implementation of targeted financial subsidies for neonatal services.

## 5. Conclusion and Recommendations

The economic burden of NICU admissions is significant, where indirect expenses, primarily food expenses, account for the largest proportion. Prolonged hospital stay and initial hospital admission were associated with higher costs. Expanding community‐based health insurance programs to cover neonatal care costs can reduce out‐of‐pocket expenditure and protect the family from financial shocks to enhance Health Insurance Coverage; promoting factors that safely support earlier discharge once a neonate is clinically ready such as strengthened follow‐up care at home and postdischarge support services can help make healthcare and family costs more affordable without compromising neonatal health outcomes. Long‐term research is needed to assess the long‐term economic impacts of NICU admissions on families and to identify whether interventions are effective in reducing financial barriers to care.

## Author Contributions

All authors were involved in the conception, design, execution, and the writing and evaluation of the work from its inception to final submission.

## Funding

No funding was received for this manuscript.

## Ethics Statement

Ethical approval was obtained from the Institutional Review Board (IRB) of the College of Medicine and Health Sciences, Hawassa University (Reference No. IRB/097/14). Prior to data collection, an official letter of cooperation was submitted to the administrative bodies of each study hospital. Verbal informed consent was obtained from all participants before administration of the questionnaire, and permission was obtained from the heads of the NICU wards at all participating hospitals. The requirement for written informed consent was waived, and verbal consent was approved by the IRB because the study was a noninterventional survey that involved no clinical procedures and posed minimal risk to participants. All data were deidentified prior to analysis. No personal identifiers, such as names or addresses, were recorded on the questionnaires; instead, each questionnaire was assigned a unique code. Only deidentified data were entered into the database and included in the analysis.

## Conflicts of Interest

The authors declare no conflicts of interest.

## Supporting Information

Additional supporting information can be found online in the Supporting Information section.

## Supporting information


**Supporting Information 1**  

**Supporting Information 2**  

## Data Availability

The data that support the findings of this study are available on request from the corresponding author. The data are not publicly available due to privacy or ethical restrictions.
