# Supplementary material for: Economic Burden of Neonatal Intensive Care Unit Admissions in Public Hospitals of Sidama Regional State, Ethiopia, 2024
Source: Int J Pediatr. 2026 Nov 15;2026:5831674. doi: 10.1155/ijpe/5831674 (PMC13770306; doi:10.1155/ijpe/5831674)
Supplement: Supplementary file 1 [file IJPE-2026-5831674-s001.pdf]

Economic Burden of Questionnaire

.

No	Question	Response
**Section A: Socio-demographic and Clinical Background (added)**		
A1	Age of parent (years)	______________ years
A2	Sex of parent	a. Male<br>b. Female
A3	Marital status	a. Single<br>b. Married<br>c. Divorced / separated<br>d. Widowed
A4	Educational level	a. No formal education<br>b. Primary<br>c. Secondary<br>d. Tertiary / above
A5	Occupation	a. Farmer<br>b. Government employee<br>c. Private employee / self-employed<br>d. Housewife<br>e. Unemployed<br>Other, specify ______________
A6	Place of residence	a. Urban<br>b. Rural
A7	Distance / travel time from home to this hospital	______________ km / ______________ hours
A8	Diagnosis or reason for this visit (if known)	______________
A9	Duration of illness, from onset of symptoms to this visit	______________ (days / weeks / months)
A10	Number of health-facility visits for this illness so far, including this one	______________ visits
**Section B: Household and Economic Background**		
1	How many household members live with the patient?	______________ people
B2	Average total monthly household income (in local currency)	______________
2	Do you have health insurance?	a. Yes<br>b. No
3	If you answered "Yes" to question 2, what type is it?	a. Community-based (CBHI)<br>b. Private / employer-based<br>Other, specify ______________
**Section C: Direct Medical Costs**		
C1	Registration / consultation fee paid for this visit	______________ (amount)
4	Did you come to the hospital for a scheduled follow-up appointment?	a. Yes<br>b. No
5	If you answered "Yes" to question 4, what was the follow-up for?	a. Drugs / medication<br>b. Laboratory tests<br>c. Imaging (e.g., X-ray)<br>d. Consultation / check-up<br>Other, specify ______________

No	Question	Response
6	Did the hospital give you a prescription for drugs or an imaging order to obtain outside the hospital?	a. Yes<br>b. No
7	Were the patient's laboratory tests done within the hospital?	a. Yes<br>b. No
8	Was the patient's imaging (e.g., X-ray) done within the hospital?	a. Yes<br>b. No
C2	Before coming to this hospital, did you visit any other provider for this illness (clinic, pharmacy, traditional healer, etc.)?	a. Yes<br>b. No
C3	If yes, how much did you spend there in total?	______________ (amount)
C4	If admitted: how many days was the patient hospitalized?	______________ days
**Section D: Indirect Costs (Patient and Caregiver Productivity Loss)**		
9	Since the patient started coming to the hospital, has he/she lost time from work?	a. Yes<br>b. No
10	How many days per month do you estimate the patient misses from work because of the illness?	______________ days
D3	Patient's average daily income/wage before the illness (if employed)	______________ (amount)
D4	How many caregivers/companions came with the patient?	______________ people
D5	Did the caregiver(s) miss work or income-generating activities to accompany the patient?	a. Yes<br>b. No
D6	If yes, how many days in total did the caregiver(s) miss?	______________ days
D7	Caregiver's average daily income/wage (if employed)	______________ (amount)
**Section E: Coping Strategies and Catastrophic Expenditure**		
11	Where did the money you spent for this come from?	a. Current income / savings<br>b. Selling goods in the market<br>c. Selling property<br>d. Borrowing from another person<br>Other, specify ______________
12	In total, how many days did you spend receiving care at this hospital, across the different departments?	______________ days
E3	Did you have to cut back on food, education, or other necessities to pay for this illness?	a. Yes<br>b. No
E4	Did the cost of this illness push your household into debt?	a. Yes<br>b. No
E5	Approximately what share of your monthly household income has this illness cost so far?	______________ % or amount

Daily Cost Summary Table — Outpatient Department

Days 1–14

		1	2	3	4	5	6	7	8	9	10	11	12	13	14
**Category**	Registration/consultation														
	Drugs														
	Laboratory														
	Imaging														

	Food														
	Transport														
	Lodging														
	Other														

Days 15–28

		**15**	**16**	**17**	**18**	**19**	**20**	**21**	**22**	**23**	**24**	**25**	**26**	**27**	**28**
**Category**	Registration/consultation														
	Drugs														
	Laboratory														
	Imaging														
	Food														
	Transport														
	Lodging														
	Other														

<u>Daily Cost Summary Table — Costs Recorded by the Patient's Caregiver/Companion</u>

Days 1–14

		**1**	**2**	**3**	**4**	**5**	**6**	**7**	**8**	**9**	**10**	**11**	**12**	**13**	**14**
**Category**	Food														
	Transport														
	Lodging														
	Other														

Days 15–28

		**15**	**16**	**17**	**18**	**19**	**20**	**21**	**22**	**23**	**24**	**25**	**26**	**27**	**28**
**Category**	Food														
	Transport														
	Lodging														
	Other														

<u>Daily Cost Summary Table — Inpatient (Hospital Admission)</u>

Days 1–14

		**1**	**2**	**3**	**4**	**5**	**6**	**7**	**8**	**9**	**10**	**11**	**12**	**13**	**14**
**Category**	Registration														
	Drugs														
	Laboratory														
	Imaging														
	Food														
	Incubator														
	Other														

Days 15–28

		15	16	17	18	19	20	21	22	23	24	25	26	27	28
**Category**	Registration														
	Drugs														
	Laboratory														
	Imaging														
	Food														
	Incubator														
	Other														
