# Supplementary material for: Economic Burden of Neonatal Intensive Care Unit Admissions in Public Hospitals of Sidama Regional State, Ethiopia, 2024
Source: Int J Pediatr. 2026 Nov 15;2026:5831674. doi: 10.1155/ijpe/5831674 (PMC13770306; doi:10.1155/ijpe/5831674)
Supplement: Supplementary file 2 [file IJPE-2026-5831674-s002.pdf]

ሀዋሳ ዩኒቨርሲቲ
ህክምናና ጤና ሳይንስ ኮሌጅ
የምርምር ስነ-ምግባር ገምጋሚ ቦርድ

HAWASSA UNIVERSITY
COLLEGE OF MEDICINE AND HEALTH SCIENCES
Institutional Review Board

Ref. No: IRB/763/26
Date: 14/07/2026

To: Dr. Achamyelesh Gebretsadik (Ph.D., Associate Professor) and Co-Authors

Subject: Confirmation of Verbal Informed Consent Approval

This is to confirm that when the Institutional Review Board (IRB) of the College of Medicine and Health Sciences, Hawassa University, reviewed the research protocol titled **"Newborn Care: Quality, Infection Prevention and Economic Burden in Sidama Regional State"** (Reference No. IRB/097/14, approved 05/12/2022), it assessed the proposed consent procedure as part of that original review.

Since the study consisted only of an interview-based survey of parents/guardians, involving no clinical procedure, intervention, or risk of harm to participants or their neonates, and all the requirements for voluntary participation, confidentiality and anonymous data collection is maintained, the IRB determined from the outset that **verbal informed consent was appropriate and sufficient**, and the requirement for written informed consent was **waived**. Verbal informed consent was to be obtained from each participating parent/guardian, as well as from the head of the NICU ward at each participating hospital, prior to administration of the study questionnaire.

This letter is provided to the authors to confirm that this consent procedure was an integral part of the original ethical approval granted for this study.

Best regards,

Embialle Mengistie Beyene
(PhD, Asso.Professor)
Institutional Review Board Chairperson

Hawassa University College of Medicine & Health Sciences Institutional Review Board

✉1560 CMHS, Hawassa-Ethiopia
